# The Oral Microbiome as Mediator between Oral Hygiene and Its Impact on Nasopharyngeal Carcinoma

**DOI:** 10.3390/microorganisms11030719

**Published:** 2023-03-10

**Authors:** Qiao-Yun Liu, Ying Liao, Yan-Xia Wu, Hua Diao, Yan Du, Yi-Wei Chen, Jin-Ru Xie, Wen-Qiong Xue, Yong-Qiao He, Tong-Min Wang, Xiao-Hui Zheng, Wei-Hua Jia

**Affiliations:** 1 School of Public Health, Sun Yat-sen University, Guangzhou 510060, China; 2 State Key Laboratory of Oncology in South China, Collaborative Innovation Center for Cancer Medicine, Guangdong Key Laboratory of Nasopharyngeal Carcinoma Diagnosis and Therapy, Sun Yat-sen University Cancer Center, Guangzhou 510060, China

**Keywords:** oral microbiome, oral hygiene, nasopharyngeal carcinoma, mediation effect

## Abstract

Oral hygiene and the alteration of the oral microbiome have been linked to nasopharyngeal carcinoma (NPC). This study aimed to investigate whether the oral microbiome plays a mediating role in the relationship between oral hygiene and NPC, and identify differential microbial taxonomies that potentially mediated this association. We conducted a case–control study that involved 218 NPC patients and 192 healthy controls. The 16S rRNA gene sequencing of the V4 region was performed to evaluate the composition of the oral microbiome. Mediation analysis was applied to explore the relationship among oral hygiene, the oral microbiome and NPC. We found that dental fillings and poor oral hygiene score were associated with increased risks of NPC (OR = 2.51 (1.52–4.25) and OR = 1.54 (1.02–2.33)). Mediation analysis indicated that dental fillings increased the risk of NPC by altering the abundance of *Erysipelotrichales*, *Erysipelotrichaceae*, *Solobacterium* and *Leptotrichia wadei*. In addition, *Leptotrichia wadei* also mediated the association between oral hygiene score and the risk of NPC. Our study confirmed that poor oral hygiene increased the risk of NPC, which was partly mediated by the oral microbiome. These findings might help us to understand the potential mechanism of oral hygiene influencing the risk of NPC via the microbiome.

## 1. Introduction

Poor oral hygiene is a major public health challenge across all ages. Oral health problems such as dental caries, periodontal disease and tooth loss affect 3.5 billion people all over the world [1]. Poor oral hygiene can affect not only the oral physiological function but also systemic health. Previous studies have found that inferior oral hygiene is a risk factor for a variety of tumors, such as oral cancer, gastric cancer, colorectal cancer, pancreatic cancer and lung cancer [2,3,4,5,6]. Studies on the association between oral hygiene status and cancer risk have primarily focused on head and neck cancers [7,8,9,10].

Nasopharyngeal carcinoma (NPC) is a head and neck malignant tumor occurring in the nasopharyngeal mucosa, which is prevalent in southern China, southeast Asia and north Africa [11,12]. According to the International Agency for Research on Cancer, there were more than 130,000 new cases of NPC worldwide in 2020, while 46.8% of those were in China [13]. NPC is a complex disease caused by Epstein–Barr virus (EBV) infection and genetic and environmental factors [14,15,16,17,18]. An epidemiological study examining the association between several indicators of oral hygiene and risk of NPC suggested that having more than three teeth filled was associated with an increased risk of NPC (OR = 1.55 (1.13–2.12)) and brushing teeth twice or more daily was inversely related to NPC risk (OR = 0.62 (0.55–0.70)) [19].

Evidence has indicated an epidemiological association between oral hygiene and NPC, but the underlying mechanisms of this association remain largely unknown. The oral microbiome, an important component of the human microbiome, can affect human health by modulating metabolism, inflammatory response and immune response [20,21,22]. Several studies have elucidated that oral hygiene status is closely related to the oral microbiome [23,24,25]. Oral pathogens such as *Streptococcus mutans*, *Porphyromonas gingivalis*, *Treponema denticola*, *Tannerella forsythia* and *Aggregatibacter actinomycetemcomitans* are strongly associated with dental caries and periodontal diseases [26,27,28]. According to recent studies, the imbalance of the oral microbial community may have an impact on NPC. A study comparing the oral microbiome of NPC patients and healthy people found that *Neisseria*, *Leptotrichia* and *Pseudomonas* were significantly enriched in the NPC group, whereas *Streptococcus* was reported to be decreased [29]. A later study reported that the overall microbial diversity of NPC patients was significantly lower in comparison to healthy controls, and the microbial structure was also different between the two groups [30]. Similarly, a recent study by our group found that the oral microbiome composition of NPC patients significantly differed from that of healthy individuals [31]. The above evidence indicated that disturbance of the oral microbiome might be one of the possible pathways for the association between oral hygiene and NPC.

Although previous studies have found associations between oral hygiene, oral microbiome and NPC, whether poor oral hygiene increases the risk of NPC through alteration of the oral microbiome is still largely unknown. Mediation analysis has been widely used in the field of epidemiology to explore the potential mechanism of risk factors affecting the occurrence and development of diseases [32]. In this study, we hypothesized that the oral microbiome is a mediator of the relationship between oral hygiene and NPC. To test this hypothesis, we established a mediation model (with oral hygiene as the independent variable, NPC as the dependent variable and oral microbiome as the mediating variable) to investigate the tripartite relationship among them. The results of this study might help us to explore the potential mechanism of poor oral hygiene increasing the risk of NPC and provide a theoretical basis for the prevention of NPC.

## 2. Materials and Methods

### 2.1. Study Design and Participants

Between February 2013 and June 2019, we recruited 218 newly diagnosed patients of NPC and 192 healthy controls from the Cancer Center of Sun Yat-sen University. The inclusion criteria of NPC cases were as follows: (1) aged 18 years or older; (2) newly diagnosed NPC patients confirmed by histopathology; (3) without any antitumor therapy before providing samples. The inclusion criteria of healthy controls were as follows: (1) aged 18 years or older; (2) no history of previous malignancies; (3) without acute or chronic infection of oral cavity, ear and nose. Informed consent was obtained from each participant prior to their enrollment in the study, and the proposal was approved by the Human Ethics Committee of Sun Yat-sen University Cancer Center (the approval numbers: GZR2013-008 and GZR2019-217).

### 2.2. Data and Saliva Sample Collection

Face-to-face interviews were conducted by well-trained investigators to collect information on demographic characteristics, smoking status, alcohol drinking and oral hygiene indicators of all subjects. Saliva samples were collected from participants during study enrollment. All participants were asked not to eat or drink for at least half an hour before providing samples. Approximately 2–3 mL saliva was collected from each participant into a 50 mL centrifuge tube, which was then divided into 2 mL sterile tubes and stored in a cryogenic refrigerator at −80 °C immediately until DNA extraction.

### 2.3. Oral Hygiene Assessment

Self-reported information on oral hygiene was recorded using interview-based questionnaires, including missing teeth after the age of 20 years, dental fillings due to caries and daily frequency of brushing teeth. An oral hygiene score was created to evaluate the association between overall oral hygiene status and risk of NPC. The oral hygiene score, ranging from 0 to 3 (with a score of 2 or more indicating poor oral hygiene), was designed to evaluate the oral health and oral hygiene habits of the subjects by summing up the following indicators: missing teeth after age 20 years (no = 0, yes = 1); filled teeth due to caries (no = 0, yes = 1); frequency of brushing teeth (≥ twice per day = 0, < twice per day = 1).

### 2.4. 16S rRNA Gene Sequencing and Bioinformatics Analysis

The DNA of the oral microbiome was extracted from saliva using the DNeasy PowerSoil kit (QIAGEN, Germany). The V4 variable region of the 16S rRNA gene was amplified using the primers 515F (5′-GTGCCAGCMGCCGCGGTAA-3′) and 806R (5′-GGACTACHVGGGTWTCTAAT-3′) and then sequenced by the MiSeq PE250 platform [33]. The sequencing data were denoised using the dada2 plugin in QIIME2 software to obtain representative sequences and feature table of the amplicon sequence variants (ASVs) [34,35]. The ASVs with summated counts of ten or fewer across all samples or present in fewer than five samples were filtered out. Taxonomic assignment of ASVs was carried out based on the trained SILVA database classification (silva-138-99-nb-classifier) [36]. The ASVs were annotated and classified into different taxonomic levels (phylum, class, order, family, genus and species), and ASVs that were unassigned or identified as Archaea were excluded from further analysis. We carried out the following bioinformatics analysis using the “phyloseq” package of R software (version 4.2.0) [37]. Samples were randomly rarefied to a depth of 5000 reads per sample. Four alpha diversity indices (observed OTUs, Chao1, Shannon and Simpson index) and the Bray–Curtis distance matrix reflecting the beta diversity were estimated based on the ASV level.

### 2.5. Statistical Analysis

We conducted a descriptive analysis to compare demographics between NPC cases and controls by using chi-square test for categorical variables and Student’s *t*-test for continuous variables. Multivariable logistic regression models were used to estimate odds ratios (ORs) and 95% confidence intervals (CIs) for associations of oral hygiene indicators with the risk of NPC. All ORs were adjusted for age, sex, educational level, cigarette smoking and alcohol drinking status. Differences with *p* < 0.05 were considered statistically significant.

We next examined associations of the oral microbiome composition with NPC, dental fillings and oral hygiene score separately. For alpha diversity, statistical differences in four alpha diversity indices (observed OTUs, Chao1, Shannon and Simpson index) between the groups were determined by the Wilcoxon rank-sum test. Beta diversity analysis was performed by principal coordinates analysis (PCoA) based on the Bray–Curtis distance. Permutational multivariate analysis of variance (PERMANOVA) with 999 permutations was conducted to test the statistical significance of the Bray–Curtis distance. For the diversity analysis, *p* < 0.05 was considered to be significant. We selected the taxa with a detection rate equal to or greater than 10% in all samples. Linear discriminant analysis (LDA) effect size (LEfSe) was applied to identify differentially abundant taxa between the groups at all taxonomic levels [38]. The threshold on the logarithmic LDA score for distinguishing microbial biomarkers was set to 2.0. False discovery rates (FDRs) were calculated using the Benjamini–Hochberg method and q-values < 0.05 were considered significant.

Finally, we conducted the mediation analysis to investigate whether the oral microbiome plays a mediating role in the association between oral hygiene and NPC. Oral hygiene-related bacteria were compared with NPC-related bacteria, and consistent bacteria were selected as mediator candidates. By referring to relevant literature, arcsine square root transformation was performed to improve the normality of the relative abundance values of mediator candidates [39,40,41]. Mediation analysis was performed using the “mediation” package in R and corrected for age, sex, educational level, cigarette smoking and alcohol drinking status. A statistical significance was confirmed as *p* value < 0.05.

All statistical analyses were performed using R software (version 4.2.0, R Core Team, Vienna, Austria).

## 3. Results

### 3.1. Characteristics of the Study Participants

The demographic characteristics of 218 NPC patients and 192 healthy controls are shown in Table 1. No statistically significant differences were observed in basic demographic variables such as age, sex, cigarette smoking and alcohol drinking between cases and controls. Compared to healthy controls, a lower percentage of NPC patients received high school education and above.

### 3.2. Associations of Oral Hygiene Factors with the Risk of NPC

To assess the associations between oral hygiene indicators and the risk of NPC, we conducted multivariable logistic regression models with or without adjusting for age, sex, educational level, cigarette smoking and alcohol drinking. Associations of NPC risk with oral hygiene factors are displayed in Table 2. In the unadjusted model, the risk of NPC was increased in subjects with tooth loss (OR = 1.50; 95% CI: 1.02–2.22), dental fillings (OR = 1.89; 95% CI: 1.18–3.05) and poor oral hygiene score (OR = 1.65; 95% CI: 1.11–2.45). Tooth brushing less than two times per day had a relatively high risk for NPC, with the OR of 1.42 (0.95–2.12). In the adjusted model, we still observed the significant associations between dental fillings and poor oral hygiene score and the increased risk of NPC, the ORs (95% CIs) for dental fillings and poor oral hygiene score were 2.51 (1.52,4.25) and 1.54 (1.02, 2.33).

### 3.3. Links between the Oral Microbiome Composition and NPC

**Figure 1:**
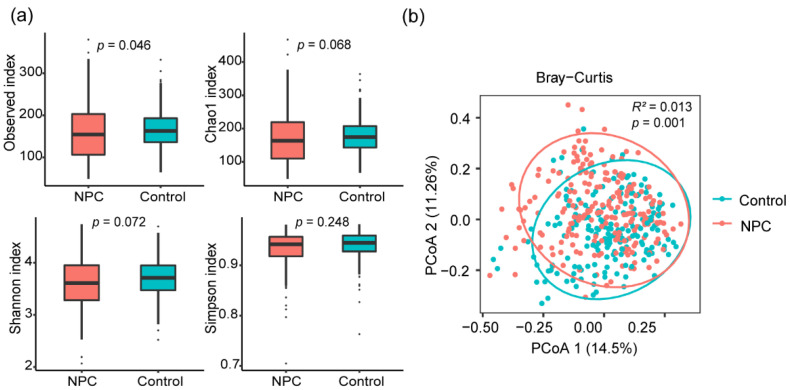
Alpha and beta diversity plots to visualize the difference in microbial community between NPC cases and controls. (**a**) Four alpha diversity indices between two groups. (**b**) Principal coordinate analysis (PCoA) based on Bray–Curtis distance of the oral microbiome between two groups.

To evaluate the association of the oral microbiome with NPC, we investigated the differences in microbiome diversity and bacterial taxa between NPC cases and healthy controls. For alpha diversity, we calculated differences in four alpha diversity indices (observed OTUs, Chao1, Shannon and Simpson index) between the two groups. We found the observed OTUs index was slightly lower (*p*  =  0.046) in the NPC group compared to the control group (Figure 1a). There were no significant associations between NPC and other alpha diversity indices (Figure 1a). For beta diversity, the results of principal coordinate analysis (PCoA) and PERMANOVA test based on the Bray–Curtis distance indicated a significant difference between the two groups (*R*^2^ = 0.013, *p* = 0.001) (Figure 1b).

Next, we assessed the differentially abundant bacterial taxa between NPC cases and controls by LEfSe analysis on the taxonomic levels. LEfSe analysis revealed that there were 97 bacterial taxa showing statistical differences between NPC cases and controls, including 3 phyla, 7 classes, 16 orders, 23 families, 28 genera and 20 species. (Table S1). Seven genera were significantly enriched in the NPC group, including *Haemophilus*, *Scardovia*, *Streptococcus*, *Gemella*, *Actinobacillus*, *Eikenella* and *Staphylococcus*. Meanwhile, 21 genera were more abundant in healthy controls, such as *Rothia*, *Solobacterium*, *Stomatobaculum* and *Lachnoanaerobaculum*.

### 3.4. Influence of Dental Fillings on Oral Microbiome Composition

**Figure 2:**
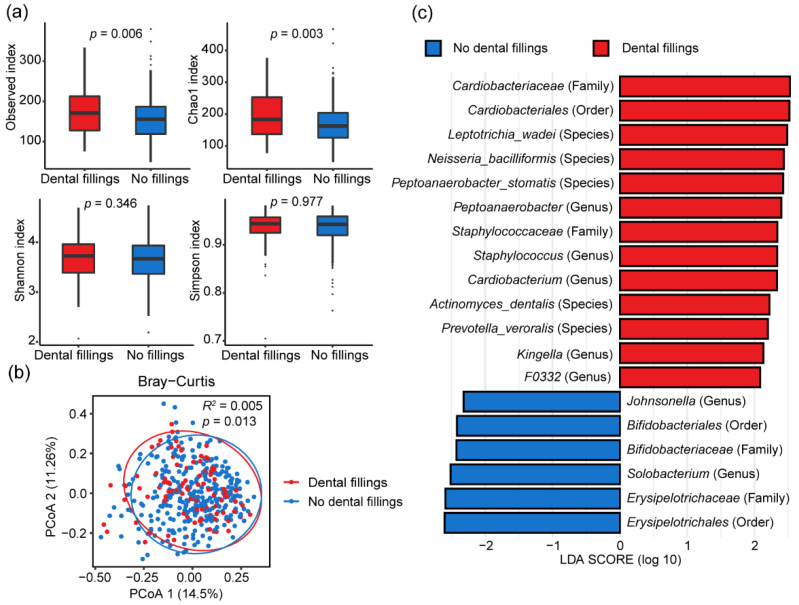
Dental fillings and the oral microbiome composition. (**a**) Four alpha diversity indices between two groups. (**b**) Principal coordinates analysis (PCoA) based on the Bray–Curtis distance of the oral microbiome between two groups. (**c**) Differentially abundant bacterial taxa between two groups according to the linear discriminant analysis effect size (LEfSe).

We next evaluated the effect of dental fillings on the alteration of the oral microbiome. We found that the alpha diversity indices of observed OTUs and Chao1 were significantly higher in subjects with dental fillings than those without (Figure 2a). We performed a principal coordinate analysis (PCoA) and PERMANOVA test based on the Bray–Curtis distance to compare whether dental fillings affected the overall microbial composition. The PERMANOVA test showed a significant difference in the oral microbial composition between the two groups (*R*^2^ = 0.005, *p* = 0.013) (Figure 2b). We performed the LEfSe analysis to identify the differentially abundant taxa between the individuals with dental fillings or not. A total of nineteen bacterial taxa were detected with LDA score > 2.0 and FDR q-value < 0.05 between the two groups (Figure 2c). Thirteen bacterial taxa were significantly enriched in the dental fillings group, such as *Staphylococcus*, *Cardiobacterium*, *Kingella*, *Leptotrichia wadei*, *Peptoanaerobacter stomatis* and *Actinomyces dentalis*, whereas six bacterial taxa were significantly less abundant in the dental fillings group, including *Bifidobacteriales*, *Erysipelotrichales*, *Bifidobacteriaceae*, *Erysipelotrichaceae*, *Johnsonella* and *Solobacterium*.

### 3.5. Associations between the Oral Microbiome Composition and Oral Hygiene Score

**Figure 3:**
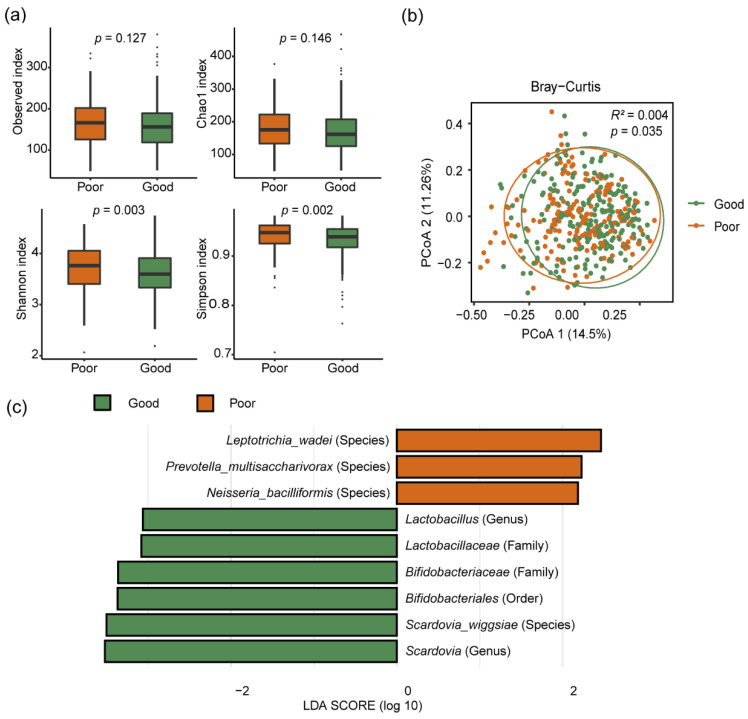
Oral hygiene score and the oral microbiome composition. (**a**) Four alpha diversity indices between two groups. (**b**) Principal coordinates analysis (PCoA) based on the Bray–Curtis distance of the oral microbiome between two groups. (**c**) Differentially abundant bacterial taxa between two groups according to the linear discriminant analysis effect size (LEfSe).

To assess the impact of overall oral hygiene status on the oral microbiome, we explored the association between oral hygiene score and the oral microbiome. For alpha diversity, we found the alpha diversity indices of Shannon and Simpson were statistically higher in the poor oral hygiene group compared to the good oral hygiene group (Figure 3a). For beta diversity, PERMANOVA analysis of Bray–Curtis distance revealed that the community structure was significantly different between the two groups (*R*^2^ = 0.004, *p* = 0.035) (Figure 3b). We identified nine bacteria that were significantly associated with oral hygiene score by LEfSe analysis. We found the abundances of *Leptotrichia wadei*, *Prevotella multisaccharivorax* and *Neisseria bacilliformis* were higher in the poor oral hygiene group compared to the good oral hygiene group, while the abundances of *Bifidobacteriales*, *Bifidobacteriaceae*, *Lactobacillaceae*, *Scardovia*, *Lactobacillus* and *Scardovia wiggsiae* were lower (Figure 3c).

### 3.6. Mediation Effects of Oral Microbiome on the Associations of Oral Hygiene with NPC

**Figure 4:**
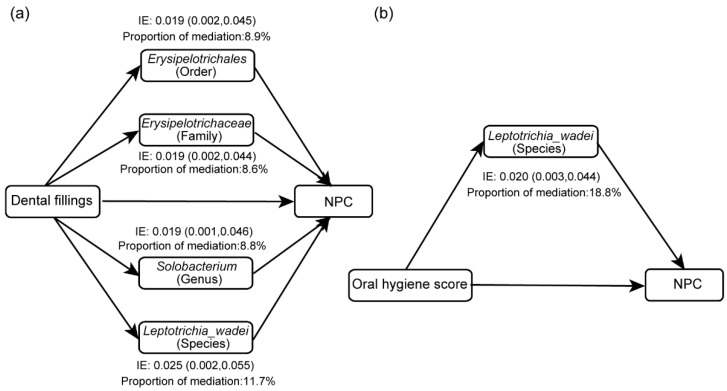
The mediating effects of potential mediators on the associations of (**a**) dental fillings and (**b**) oral hygiene score with the risk of NPC. (IE: indirect effect).

To investigate whether oral hygiene affects NPC through the oral microbiome, we performed mediation analyses adjusting for age, sex, educational level, cigarette smoking and alcohol drinking. Based on the above results, we found that the relative abundances of *Erysipelotrichales*, *Erysipelotrichaceae* and *Solobacterium* were significantly negatively associated with both dental fillings and NPC, and *Staphylococcaceae*, *Staphylococcus*, *Leptotrichia wadei* and *Neisseria bacilliformis* were positively associated with dental fillings and NPC. We took the seven bacteria as candidate mediators to explore the mediating effects in the relationship between dental fillings and NPC, respectively. Mediation analysis showed that *Erysipelotrichales*, *Erysipelotrichaceae*, *Solobacterium* and *Leptotrichia wadei* partially mediated the association between dental fillings and risk of NPC, and the corresponding mediating effects were 8.9%, 8.6%, 8.8% and 11.7%, respectively (Figure 4, Table S2). Similarly, we observed that *Leptotrichia wadei* and *Neisseria bacilliformis* were enriched in both the poor oral hygiene group and the NPC group, so they were regarded as potential mediators in further mediation analysis. The mediation analysis indicated that the association of oral hygiene score with NPC was partially mediated by *Leptotrichia wadei*, and *Leptotrichia wadei* explained 18.8% of the total impact of oral hygiene score on risk of NPC (Figure 4, Table S2). The mediation effects of the oral microbiome on the associations between oral hygiene and NPC are provided in Table S2.

## 4. Discussion

In this study, we investigated the relationship between oral hygiene, the oral microbiome and NPC. We found that subjects with dental fillings and poor oral hygiene score were associated with an increased risk of NPC and identified oral bacterial taxa including *Erysipelotrichales*, *Erysipelotrichaceae*, *Solobacterium* and *Leptotrichia wadei* that potentially mediated these associations. Our study highlighted the association between poor oral hygiene and the increased risk of NPC, and revealed the mediating effects by the alteration of the oral microbiome.

Previous studies have reported the association between oral hygiene and the risk of NPC. A hospital-based case–control study in Turkey reported that less frequent tooth brushing and more dental caries were significantly associated with an increased risk of NPC [42]. Another population-based case–control study in southern China found that more dental fillings were linked to higher NPC risk, whereas brushing teeth twice or more daily was associated with a decreased risk of NPC [19]. The results of our study confirmed that poor oral hygiene could lead to an increased risk of NPC. In the present study, we found that having dental fillings significantly increased NPC risk. In addition, to comprehensively evaluate the impact of oral hygiene on NPC, oral hygiene score was constructed by summing up three oral hygiene indicators, including tooth loss, dental fillings and frequency of brushing teeth, and the result indicated that poor oral hygiene score was a risk factor for NPC.

The biological mechanism by which poor oral hygiene increases the risk of NPC remains unclear. Inflammation might be one of the important components of the carcinogenesis process. Periodontal disease can cause low-grade systemic inflammation, which can elevate the level of inflammatory mediators such as IL-1, IL-6 and C-reactive protein [43]. Another possible mechanism is that poor oral hygiene causes carcinogenic effects through the oral microbiome. First, periodontal pathogens have been reported to exert potential carcinogenesis by producing carcinogenic metabolites. For example, the accumulation of *Veillonella* and *Actinomyces* spp. can promote the conversion of nitrate to nitrite, which might assist in the formation of carcinogenic nitrosamines [44,45]. Oral bacteria, such as some *Streptococcus* species and *Neisseria* species, are reported to catalyze the conversion of ethanol to acetaldehyde in saliva, which might increase the risk of oral cancer [46]. Second, bacteria might affect the EBV, one of the causes of NPC. EBV has been reported to co-infect with *Porphyromonas gingivalis* in the oral cavity of periodontal patients [47,48]. Researchers also found that *Porphyromonas gingivalis* can activate the EBV latent in cells by secreting butyric acid [49]. Furthermore, the disturbance of the oral microbiome may indirectly promote the occurrence and development of cancers by causing persistent chronic inflammation and suppressing immune response [50,51]. Several studies have shown that the periodontal pathogen *Fusobacterium nucleatum* might increase the risk of head and neck cancers by indirect carcinogenic effects [52,53]. A recent study revealed that CD8^+^ T infiltration was decreased in NPC tumor tissues with high bacterial load, indicating that the microbiome may influence NPC by modulating the immune microenvironment [54].

We found that the alpha diversity showed a decreasing trend in the NPC group. A previous study has also found that the alpha diversity was lower in the NPC cases than in controls [30]. We identified some bacteria whose abundances were significantly different between NPC cases and controls. For example, *Staphylococcus*, *Gemella* and *Streptococcus* were enriched in the NPC group, while the abundances of *Rothia*, *Lachnoanaerobaculum* and *Stomatobaculum* were less abundant. A recent study on the relationship between the intratumoral microbiome and the prognosis of NPC found that high abundance of *Staphylococcus* was apparent in the NPC tumor tissues [54]. *Gemella* has been reported to be a microbial marker for some digestive system tumors [55,56]. High abundance of Streptococcus was positively associated with the risk of esophageal squamous cell carcinoma [57]. *Rothia*, a normal microbiome of the human mouth, was generally associated with good oral health [58,59], and has been reported to be less abundant in patients with oral cancer [60]. A study assessing the relationship between the viral copy numbers of EBV and the abundances of salivary microbes found that the abundances of *Lachnoanaerobaculum* and *Stomatobaculum* were negatively correlated with the viral copy numbers of EBV [61]. Furthermore, a meta-analysis showed that *Lachnoanaeraculum* exhibited a significant decrease in lung tumor tissues compared with normal tissues [62].

We also explored the relationship between oral hygiene and the oral microbiome. Our study revealed that higher alpha diversity was found in the poor oral hygiene group, which was consistent with previous studies [24,63]. We identified microbiome biomarkers of dental fillings and poor oral hygiene score, some of which have been reported to be harmful to oral health in previous studies. *Staphylococcus* spp. is one of the common bacterial genera in periodontitis and can be isolated in all stages of periodontitis [64]. *Peptoanaerobacter stomatis* is an emerging periodontal pathogen that can drive proinflammatory processes by affecting neutrophils [65,66]. *Actinomyces dentalis*, a Gram-positive bacterium originally isolated from human dental abscess [67], has been reported to be enriched in patients with periodontitis [68]. *Prevotella multisaccharivorax* has previously been reported to be associated with severe caries [69,70]. *Neisseria bacilliformis* was found to cause opportunistic infections of the oral cavity and respiratory tract [71].

Our study found that *Erysipelotrichales*, *Erysipelotrichaceae*, *Solobacterium* and *Leptotrichia wadei* partially mediated the effect of dental fillings on risk of NPC. *Leptotrichia wadei* also mediated the association of poor oral hygiene score with an increased risk of NPC. Previous research revealed that the relative abundance of *Erysipelotrichaceae* was negatively correlated with the risk of lung cancer [72]. A case–control study exploring the oral microbiome and colorectal cancer found that *Erysipelotrichaceae* and *Solobacterium* were associated with a reduced risk of colorectal cancer [73]. Crohn’s disease, an inflammatory bowel disease, has been found to be linked to the decreased abundance of *Erysipelotrichaceae* [74,75]. We conjectured that *Erysipelotrichaceae* might affect the level of inflammation in the body. *Leptotrichia wadei* was found to be more frequent in subjects with dental caries compared to healthy individuals [76] and was also associated with halitosis [77]. The increased abundance of *Leptotrichia wadei* was associated with high risk of gastric cancer [78]. The genus *Leptotrichia* has been reported to be more significantly abundant in saliva samples from NPC patients than in healthy controls [29]. A study speculated that *Leptotrichia* may promote carcinogenesis by influencing the immune–inflammatory response [79]. Our results support associations between these taxa and NPC, but the detailed mechanisms in the carcinogenesis process are not clear yet. To verify these associations, additional studies that investigate how these bacteria are involved in the occurrence and development of NPC might be valuable.

It is well known that EBV infection is closely related to the pathogenesis of NPC. The relationship between the oral microbiome and EBV in the development of NPC deserves further investigation. Although the evidence of interaction between EBV and the bacteria reported in this study are still lacking, the bacteria have previously been reported to be associated with viral infections. *Leptotrichia* spp. was found to coexist with HPV in head and neck cancer patients, which suggested the role of bacterial and viral co-infection in the carcinogenesis process [80]. Previous studies have also found some evidence of EBV interacting with other oral bacteria. Co-existence of EBV and the pathogenic bacterium *Porphyromonas gingivalis* was significantly higher in periodontitis patients than in healthy controls [47,48]. This hints that bacteria may co-operate with EBV in the process of carcinogenesis. On the other hand, the bacteria can reactivate the latent EBV and increase the free EBV load. A recent study on the oral microbiome in NPC by our team provided clues to the indirect etiological role of *Streptococcus sanguinis* in promoting EBV lytic activation in EBV-positive cells [31].

To our knowledge, this is the first study to evaluate the role of the oral microbiome in mediating the association between oral hygiene and risk of NPC. However, we note that the study has some limitations. Firstly, our study was a cross-sectional study, so we could not characterize the causal relationships between oral hygiene, oral microbiome and NPC. Secondly, the relatively small sample size of this study limited the statistical power of the analysis, and the findings need to be further verified in a large sample size population. In addition, the oral hygiene information we collected was self-reported, which might introduce reporting bias. Finally, EBV infection is closely linked to the emergence of NPC, but this study did not detect the relevant indicators of EBV infection. The interaction between the oral microbiome and EBV in the development of NPC needs to be explored further.

## 5. Conclusions

This study suggested that having dental fillings and poor oral hygiene score may increase the risk of NPC, which emphasized the importance of keeping good oral health. Furthermore, the alteration of the oral microbiome partially mediated the association between oral hygiene and NPC. These findings extend our understanding of the mechanism by which poor oral hygiene increases the risk of NPC.

## Figures and Tables

**Table 1:** Characteristics of nasopharyngeal carcinoma (NPC) patients and controls.

Characteristics	NPC (*n* = 218)	Control (*n* = 192)	*p*-Value
Age, years (mean ± SD)	48.33 (10.07)	46.53 (10.33)	0.075 ^a^
Sex, *n* (%)			0.876 ^b^
Male	157 (72.02)	136 (70.83)	
Female	61 (27.98)	56 (29.17)	
Educational level, *n* (%) ^c^			0.009 ^b^
<High school	178 (81.65)	134 (70.16)	
≥High school	40 (18.35)	57 (29.84)	
Cigarette smoking, *n* (%)			0.119 ^b^
Never	85 (38.99)	89 (46.35)	
Former	32 (14.68)	17 (8.85)	
Current	101 (46.33)	86 (44.79)	
Alcohol drinking, *n* (%)			0.915 ^b^
Non-drinker	152 (69.72)	132 (68.75)	
Drinker	66 (30.28)	60 (31.25)	

^a^: *p*-values were based on Student’s *t*-test. ^b^: *p*-values were based on Pearson’s chi-squared test. ^c^: Numbers do not correspond to the total sample size due to missing values.

**Table 2:** Odds ratios (ORs) and 95% confidence intervals (CIs) of nasopharyngeal carcinoma associated with oral hygiene.

Oral Hygiene	NPC *n* (%)	Controls *n* (%)	OR (95% CI) ^a^	*p*-Value ^a^	OR (95% CI) ^b^	*p*-Value ^b^
Tooth loss				0.042		0.207
No	95 (43.58)	103 (53.65)	1.00		1.00	
Yes	123 (56.42)	89 (46.35)	1.50 (1.02, 2.22)		1.32 (0.86, 2.02)	
Dental fillings				0.008		<0.001
No	155 (71.10)	158 (82.29)	1.00		1.00	
Yes	63 (28.90)	34 (17.71)	1.89 (1.18,3.05)		2.51 (1.52, 4.25)	
Tooth brushing				0.091		0.442
≥2 times per day	71 (32.57)	78 (40.62)	1.00		1.00	
<2 times per day	147 (67.43)	114 (59.38)	1.42 (0.95, 2.12)		1.18 (0.77, 1.81)	
Oral hygiene score				0.013		0.040
Good (0–1)	106 (48.62)	117 (60.94)	1.00		1.00	
Poor (2–3)	112 (51.38)	75 (39.06)	1.65 (1.11, 2.45)		1.54 (1.02, 2.33)	

^a^: Unadjusted. ^b^: Adjusted for age, sex, educational levels, cigarette smoking and alcohol drinking.

## Data Availability

The data presented in this study are available from the corresponding author on reasonable request.

## Supplementary Materials

The following supporting information can be downloaded at: https://www.mdpi.com/article/10.3390/microorganisms11030719/s1, Table S1: Differentially abundant taxa identified by LEfSe between nasopharyngeal carcinoma cases and controls; Table S2: The mediation effects of potential mediators on the associations of oral hygiene with the risk of NPC.
